# Does the inclusion of 'professional development' teaching improve medical students' communication skills?

**DOI:** 10.1186/1472-6920-11-41

**Published:** 2011-06-27

**Authors:** Katherine Joekes, Lorraine M Noble, Angela M Kubacki, Henry WW Potts, Margaret Lloyd

**Affiliations:** 1 Division of Medical Education, UCL, Archway Campus, Highgate Hill, London, N19 5LW, UK; 2 Division of Population Health Sciences and Education, St George's, University of London, Hunter Wing, Cranmer Terrace, London, SW17 0RE, UK; 3 Centre for Health Informatics and Multiprofessional Education, UCL, Archway Campus, Highgate Hill, London, N19 5LW, UK; 4 Research Department of Primary Care and Population Health, UCL, Royal Free Campus, Rowland Hill Street, London, NW3 2PF, UK

**Keywords:** communication skills, patient-centredness, medical student, curriculum change, video observation

## Abstract

**Background:**

This study investigated whether the introduction of professional development teaching in the first two years of a medical course improved students' observed communication skills with simulated patients. Students' observed communication skills were related to patient-centred attitudes, confidence in communicating with patients and performance in later clinical examinations.

**Methods:**

Eighty-two medical students from two consecutive cohorts at a UK medical school completed two videoed consultations with a simulated patient: one at the beginning of year 1 and one at the end of year 2. Group 1 (n = 35) received a traditional pre-clinical curriculum. Group 2 (n = 47) received a curriculum that included communication skills training integrated into a 'professional development' vertical module. Videoed consultations were rated using the Evans Interview Rating Scale by communication skills tutors. A subset of 27% were double-coded. Inter-rater reliability is reported.

**Results:**

Students who had received the professional development teaching achieved higher ratings for use of silence, not interrupting the patient, and keeping the discussion relevant compared to students receiving the traditional curriculum. Patient-centred attitudes were not related to observed communication. Students who were less nervous and felt they knew how to listen were rated as better communicators. Students receiving the traditional curriculum and who had been rated as better communicators when they entered medical school performed less well in the final year clinical examination.

**Conclusions:**

Students receiving the professional development training showed significant improvements in certain communication skills, but students in both cohorts improved over time. The lack of a relationship between observed communication skills and patient-centred attitudes may be a reflection of students' inexperience in working with patients, resulting in 'patient-centredness' being an abstract concept. Students in the early years of their medical course may benefit from further opportunities to practise basic communication skills on a one-to-one basis with patients.

## Background

The General Medical Council states that doctors have a duty to 'work in partnership with patients' [1], combining effective clinical communication skills with an attitude towards patients which is respectful and supportive. The national core curriculum for communication skills in undergraduate medical education [2] similarly notes that respect for patients is a fundamental attitude which must underpin the teaching of communication skills. This guidance is the result of accumulated evidence that the relationship between the patient and doctor is closely linked to improved patient outcomes, such as patient satisfaction [3] and adherence to medical recommendations [4], as well as patient understanding and recall, and symptom resolution [5].

It has been established that certain important clinical communication skills do not develop spontaneously with exposure to clinical environments [6]. A combination of didactic and experiential learning, however, can foster effective clinical communication skills in medical students [7,8] and qualified clinicians [9,10]. However, the relationship between communication skills and attitudes towards patients is not entirely clear [11]. There is an increased focus on teaching interventions to foster professional and patient-centred attitudes at medical school [12]. However, there is a need to determine whether this affects professional attitudes as well as behaviours (such as improved communication skills), particularly as patient-centred attitudes are known to decline with increased clinical experience [13]. Furthermore, studies have found that clinicians' confidence in their clinical skills (including communication skills) is not necessarily congruent with observed skills [14]. Therefore, it is important to determine whether any interventions have both immediate and long-lasting effects: if improvements in communication skills and patient-centred attitudes are achieved early in medical training, are these maintained over the entire course?

There is a further question about whether communication skills teaching is most effective when delivered as 'stand-alone' teaching, where the focus is specifically on 'teaching communication skills', or as part of integrated teaching (e.g. in case-based teaching) which addresses a number of domains within a session [15]. While stand-alone communication skills teaching is known to be effective in improving observable communication skills [6], it risks creating a 'silo effect', which may cause the learner difficulty in generalising the knowledge and skills [16]. The impact on communication behaviour of integrated teaching requires further exploration.

Measurement of students' observed communication skills has been achieved in previous research using behavioural rating of videoed consultations with standardised patients. Studies of medical students [17-20] and doctors [21,22] have concluded that differences in communication behaviours after an intervention can be identified using video recordings. Furthermore, this method is sufficiently sensitive to detect differences in communication 'process' skills, such as effective information gathering [19] and non-verbal skills [23] in medical student consultations.

The current study is part of a prospective investigation examining the effects of introducing early professional development teaching into a UK medical curriculum [24]. This included communication skills teaching as part of a vertical module in the first two years of undergraduate medical training. However, rather than the focus being on behavioural skills training, communication skills teaching was placed in the context of integrated, case-based teaching, addressing a number of domains, including professional attitudes. This study examines the effect of professional development training on students' observed communication skills, and the relationship between observed communication, confidence in communicating with patients, patient-centred attitudes, and performance in later clinical examinations.

### Aims of the study

(1) To determine whether the introduction of professional development teaching in the first two years of the medical course improved students' observed communication skills, by comparing students from two consecutive cohorts at a UK medical school.

(2) To establish whether students' patient-centred attitudes and confidence in communicating with patients were related to observed communication skills.

(3) To determine whether observed communication skills in early medical training (years 1 and 2) are related to performance in later clinical assessments (objective structured clinical examinations) (years 3 and 5).

## Methods

### Participants

The participants were undergraduate medical students in two consecutive cohorts, invited to participate in their first week at a UK medical school. Cohort 1 received a traditional pre-clinical curriculum in the first two years. Cohort 2 received a curriculum in the first two years which included communication skills teaching integrated into a vertical module called 'professional development'. All students in both cohorts were invited to participate in a questionnaire-based study (n = 626 in total, comprising 306 in the traditional curriculum and 323 in the professional development curriculum). The sample was 59% female (n = 270), with a mean age of 19.0 years at baseline (standard deviation 2.0, range 17-31 years). The ethnic composition of the sample was 47% White, 35% Asian (Indian subcontinent), and 19% Other ethnic groups.

A subsample of 70 students from each cohort (22%) was invited to take part in a videoed interview with a simulated patient, which represented the capacity of the communication skills suite to video students within their first week at medical school.

### Design

This was a longitudinal, prospective study. Students were not allocated to cohorts, but entered medical school via normal admissions procedures. Students invited to participate in the videoed interviews were randomly selected from their respective cohorts. Data collection took place in the first week of year 1, prior to the first teaching session in professional development, and after the end of teaching in year 2.

### Teaching Intervention

In the traditional course, the curriculum in the first two years focused on basic medical sciences, taught using lectures and practical work. A small amount of small group communication skills teaching was included, which included role play and a visit to a community-based patient. This was not integrated with other aspects of the course. Following the pre-clinical course, students attended the clinical programme for three years. Preparation for the clinical and professional aspects of the students' role in the clinical programme was undertaken in the introduction to students' clinical attachments.

The new curriculum was designed to increase the focus on students learning professional and clinical skills early in the medical course, to better prepare them for their clinical programme, and to integrate the learning of professional and clinical skills with the basic medical sciences. The vertical module called 'professional development' integrated communication skills, practical clinical skills, ethics and law, health promotion, community-oriented medicine and evaluation of evidence. Its core aim was to enable students to acquire the professional knowledge, skills and attitudes necessary for the practice of medicine. Students attended for one morning a week each year. Teaching methods included lectures, small-group seminars, meeting patients and one simulated patient, and visits to community health facilities. Groups of approximately 15 students were facilitated by 24 tutors in each year using centrally prepared lesson plans, tutor guides and student course books. Tutors were trained in the delivery of the teaching materials. Links were made between basic medical sciences teaching and the professional development teaching by means of case scenarios related to each teaching module.

### Measures

#### Questionnaire

• Demographic data (gender, age, ethnicity, matriculation status)

• Patient-centred attitudes: a 17-item, shortened version of the Doctor-Patient (DP) Scale [24,25] was used. The measure comprises three subscales: (i) 'holistic care' (8 items), which indicates to what extent the student would take account of the patient's feelings and perspective; (ii) 'complexity of care' (5 items), which indicates an attitude that medical care is complex and difficult for patients to understand, (iii) 'patient decision-making' (4 items) which indicates the importance given to the patient's involvement in decision making. Items were scored from 1 (*strongly agree*) to 5 (*strongly disagree*). Higher scores indicated a more patient-centred approach.

• Confidence in communicating: 4 items were used to assess reported confidence in the ability to communicate: *I feel confident in communicating with patients, I feel I know how to listen to patients, I feel I know what to say to patients, I feel I can understand what it is like to be ill*. Items were scored from 1 (*totally disagree) *to 5 (*totally agree*). Higher scores indicated greater confidence. A 7-item scale assessed nervousness about communicating with patients [24]. Items were scored from 1 (*totally disagree*) to 5 (*totally agree*). Higher scores indicated greater nervousness.

#### Observed communication skills

• The 16-item Interview Rating Scale [17] was used to assess generic communication skills (e.g. opening and closing the interview, establishing rapport with the patient, appropriate questioning style, eye contact). These skills were not dependent on the clinical scenario or students' medical knowledge. Items were scored from 1 (*poor*) to 4 (*very good*). The scoring instructions include precise behavioural criteria for each of the four points for each item. One item (seating arrangement) was excluded, as this was pre-set in the communication skills suite.

#### Performance assessments

• Students completed a summative objective structured clinical examination (OSCE) at the end of years 3 and 5. The examinations included practical skills and consultations with real and simulated patients. Results were gathered over three consecutive academic years, to allow data from all the participating students to be collected. During this time the format of the OSCEs was comparable, although there was variation in the scenarios used. Total examination marks (expressed as percentages) were used.

### Procedure

#### Data collection

##### Questionnaires

Students completed the questionnaires immediately after a general introductory lecture in Week 1. Matched sets of questionnaire data were obtained from 454/626 eligible students: 199 from the traditional curriculum and 255 from the professional development curriculum. Non-participation was mainly due to student non-attendance at the introductory lecture in year 1 and the final lecture in year 2, due to a variety of reasons.

##### Videoed consultations

Students invited to participate in the videoed interviews were allocated appointments at the communication skills suite during the first week of term and paid £3 travel expenses. On arrival, students were given the following instruction: *'This patient has a health problem at the moment. We would like you to ask about this problem and the impact it has on their life, and also to find out more about the person.' *Students were told that the interview should take approximately 10 minutes. Students interviewed a single simulated patient on each occasion, in alignment with the teaching experience during the first year. A book token worth £10 was offered as an incentive to return for the second interview.

A total of 198 videoed interviews were obtained: 115 students completed videoed interviews in year 1 (52 traditional curriculum, 63 professional development curriculum) and 83 in year 2 (36 traditional curriculum, 47 professional development curriculum). Non-attendance in year 1 was mainly due to students starting the medical course late (after Week 1), students expected on the medical course who did not begin the course, or an unanticipated clash in the student's introductory appointment with their academic tutor. Non-attendance in year 2 was mainly due to students not attending the final teaching sessions, having left the course or having taken an interruption of study. One interview was discarded due to poor audio quality.

This resulted in a data set of 82 students providing video interviews in both years 1 and 2 (35 traditional curriculum, 47 professional development curriculum). The students providing the data were 59% female (n = 53) and with a mean age of 19.0 years. There were no differences between the student characteristics of the students providing video data compared to the students providing questionnaire data.

#### Rating of observed communication

The videoed interviews were rated by nine communication skills tutors with health care backgrounds. Each consultation was rated once and a subset of 27% (n = 53) were double-coded by a second rater. Seven raters took responsibility for the first ratings. Training comprised practice with student consultations and comparison of ratings. The seven raters were given a mean of 24 consultations each (range 6-34, median 27). Two raters were involved in the second rating. These raters trained together and conducted reliability checks on each batch of consultations that were double-coded. Thirty three consultations were rated by both second raters during training and reliability checks, and a further 20 consultations were rated by a single second rater. Raters were blind to student cohort and year of the course.

#### Inter-rater reliability

For the individual items on the Interview Rating Scale, inter-rater reliability was calculated by weighted kappa (κ) [26] as the items were rated on a 4-point scale. This identified three items as having 'moderate' agreement (0.4 < κ < 0.6), nine items as having 'fair' agreement (0.2 < κ < 0.4) and three items as having a 'poor' agreement (κ < 0.2) [27].

The three items with poor agreement were picking up leads, responding to psychosocial concerns and clarity. In their comments about the rating process, the raters noted that it was difficult to rate the first two of these items if there were no particular leads or concerns raised by the simulated patient. Similarly, the raters noted that 'clarity' was difficult to rate if the simulated patient did not make any statements which were ambiguous, or if the students did not use any medical jargon, as there was no reason for the student to take specific steps to improve clarity of the discussion. These items were excluded from the total score and from further analyses (Table 1).

**Table 1:** Inter-rater reliability of individual Interview Rating Scale items (n = 53)

	Item	Weighted kappa (κ)
1	Beginning of interview	0.54
2	Body posture	0.21
3	Eye contact	0.47
4	Frequency of interruptions	0.29
5	Use of silence	0.23
6	Use of facilitation	0.42
7	Ability to keep discussion relevant	0.36
8	Picking up leads	0.17^§^
9	Covering psychosocial concerns	0.16^§^
10	Discussion of personal issues	0.22
11	Empathy	0.25
12	Warmth	0.22
13	Questioning style	0.21
14	Clarity	0.11^§^
15	Ending of interview	0.24

^§^Three items with a poor weighted kappa (κ < 0.20), items 8, 9 and 14, were excluded from further analyses [25].

Inter-rater reliability for the Interview Rating Scale total score (based on 12 items) was assessed using the Bland-Altman method [28], which allows comparison of two subjective measurements or between scores consisting of continuous data. The possible total score ranges between 12 and 48. The difference between the scores of the first and second raters was plotted against their mean. This plot identified one rater as being consistently more generous than the second raters, indicating that the rater had not used the full range of rating criteria as instructed. This rater was excluded and the consultations were rated by a second rater. The mean bias between scores from the first and second rating was 0.47, with 95% limits of agreement between 10.15 and 11.09. This indicates that the score by the first rater may be 10 points below or 11 points above the second rater's scores, i.e. raters would differ at most by 1 point per item. This was deemed to be acceptable for the purposes of this study. Where videos had been double-rated, the mean was calculated and used as final score.

### Analyses

Comparison of cohorts was conducted using repeated measures ANOVA. The relationships between observed communication skills, confidence in communicating with patients, patient-centred attitudes, and the clinical exam (OSCE) results were explored using Pearson's correlations (relationships between variables at a single point in time) and partial correlations (relationships in year 2, controlling for scores in year 1). As an indication of power, given two groups of 36 and 47 participants, a t-test would have 80% power to detect a difference of 0.63 standard deviations between two groups, which represents a difference of 3.2 on the Interview Rating Scale.

### Ethics

The study was approved by UCL Ethics Committee.

## Results

### The impact of professional development teaching on observed communication skills

A repeated measures ANOVA was performed with total Interview Rating Scale score as the dependent variable, a within-subject factor of time (year 1 and year 2) and a between-subject factor of cohort (traditional and professional development). There was a significant effect of time (*F*_1,81 _= 30.9, *p *< 0.001), with students improving in observed communication skills over time, but not of cohort (*F*_1,81 _= 2.1, *p *= 0.15) or of the interaction between time and cohort (*F*_1,81 _= 1.6, *p *= 0.20).

The Interview Rating Scale consists of 12 items assessing different basic communication skills that may respond differently to a teaching intervention or change differently over time. To investigate this, a global test was performed, treating students' item scores as a 12-dimensional vector. This matches the repeated measures ANOVA above, but with an additional within-subject factor of item (with 12 levels). This showed a significant three-way interaction between time, cohort and item (*F*_11,45 _= 2.4, *p *= 0.02). This indicated that scores vary over time, between the two cohorts and depending on the Interview Rating Scale item involved, and that the variation in scores over each of those factors depended on the other two factors.

To explore the nature of this variation, a set of post-hoc tests was performed, 12 repeated measures ANOVAs (one for each item), each with a within-subject factor of time and a between-subject factor of cohort (Table 2). As a set of post-hoc tests, no adjustment for multiple testing was made. If a Bonferroni correction were used, a cut-off of p < 0.0042 would apply and some of the results would not achieve statistical significance.

**Table 2:** Mean scores by time and cohort for Interview Rating Scale (total and individual items)

Interview Rating Scale scores	Traditional curriculum		Professional development curriculum		Repeated measures ANOVA^§^
	**Year 1**	**Year 2**	**Year 1**	**Year 2**	

Total score	30.70	33.64	31.06	35.80	Time: *F*_1,81 _= 30.9, *p *< 0.001
*Items:*					
Beginning of interview	1.95	2.32	1.97	2.50	Time: *F*_1,63 _= 11.1, *p *= 0.001
Body posture	2.91	2.97	2.74	3.23	Time: *F*_1,81 _= 5.5, *p *= 0.021
Eye contact	3.40	3.76	3.67	3.82	Time: *F*_1,81 _= 11.4, *p *= 0.001
Frequency of interruptions	2.87	2.92	2.98	3.39	Time: *F*_1,81 _= 4.4, *p *= 0.039 Cohort: *F*_1,81 _= 4.5, *p *= 0.036
Use of silence	2.29	2.42	2.61	2.99	Time: *F*_1,80 _= 4.6, *p *= 0.035 Cohort: *F*_1,80 _= 7.5, *p *= 0.004
Use of facilitation	2.72	2.94	2.95	3.12	Time: *F*_1,80 _= 4.6, *p *= 0.035
Ability to keep discussion relevant	3.19	3.30	2.70	3.69	Interaction: *F*_1,81 _= 18.0, *p *< 0.001
Discussion of personal issues	2.03	2.38	2.27	2.57	Time: *F*_1,74 _= 4.1, *p *= 0.047
Empathy	2.20	2.40	2.07	2.34	no effects significant
Warmth	2.77	2.84	2.70	3.01	no effects significant
Questioning style	2.36	2.84	2.44	2.87	Time: *F*_1,81 _= 17.0, *p *< 0.001
Ending of interview	1.99	2.37	1.91	2.16	Time: *F*_1,81 _= 8.2, *p *= 0.005

^§ ^Significant effects only shown; main effects are not shown if the interaction was significant.

For most items, as with the total score, there is only a significant main effect of time, with scores increasing between year 1 and year 2. However, empathy and warmth showed no significant changes. With frequency of interruptions and use of silence, there was a significant main effect of time, but also of cohort, with students receiving the professional development curriculum scoring higher. For ability to keep the discussion relevant, there was a significant interaction, with students receiving the traditional curriculum showing little change between the two times, but students receiving the professional development curriculum showing a large increase.

### Interview Rating Scale and student characteristics

In year 1, Interview Rating Scale scores for female students (mean = 31.83, *SD = 4.88*, n = 53) were higher than male scores (mean = 29.22, *SD = 5.73*, n = 29) (*t *= 2.17, *p *= 0.03). This difference remained in year 2, where female students (mean = 35.96, *SD = 4.96*, n = 53) again scored higher on the Interview Rating Scale than males (mean = 32.90, *SD = 4.67*, n = 29) (*t *= 2.71, *p *= 0.01). There was no difference between students who had entered the medical course immediately after leaving school compared to students who entered medical school later, nor was there a relationship between student age or ethnic group and Interview Rating Scale score.

### Relationship between observed communication skills, confidence in communicating with patients and patient-centred attitudes

No relationship was found between total Interview Rating Scale score in year 1 and students' self-reported confidence or nervousness in talking to patients.

In year 2, significant relationships were found between total Interview Rating Scale score and students' confidence in knowing how to listen to patients and nervousness in talking to patients (Table 3).

**Table 3:** Raw and partial correlations between Interview Rating Scale total scores and students' confidence and nervousness in communicating with patients (year 2)

	Raw correlation (r)	Partial correlation (r)
Confidence in communicating with patients	0.11	0.08
Knowing how to listen to patients	0.21	0.23*
Knowing what to say to patients	0.10	0.07
Understand being ill	-0.11	-0.16
Nervousness in talking to patients	-0.25*	-0.26*

The partial correlation controlled for score on Interview Rating Scale in year 1.

* p < 0.05

There were no differences in the relationships between Interview Rating Scale score and confidence and nervousness between the two cohorts. Furthermore, no relationships were found between total Interview Rating Scale score and patient-centred attitudes in year 1 or in year 2.

### Relationship between observed communication skills and later performance in OSCEs

There was no relationship between Interview Rating Scale scores in years 1 or 2 and students' year 3 clinical examination (OSCE) results.

In year 5, some students had been lost to the study (due to re-sitting examinations, interruptions of study or leaving medical school). This resulted in a reduced sample size (n = 73). A significant inverse relationship was identified between the Interview Rating Scale total scores in year 1 and the overall year 5 OSCE scores for traditional curriculum students (*r *= -0.36, *p *= 0.040, n = 33). No such relationship was found for professional development curriculum students.

## Discussion

These findings indicate that all students improved in observed communication skills with simulated patients over the first two years at medical school. There was a trend for the students receiving professional development teaching to perform better overall than traditional curriculum students at the end of the second year. Students receiving professional development teaching were judged to be better at using silence, not interrupting the patient and keeping the discussion relevant. Few relationships were found between students' observed communication skills, their confidence in talking with patients, and their patient-centred attitudes. Furthermore, the only relationship between observed communication skills at the start of medical training and performance in final year clinical examinations was an inverse relationship for the traditional curriculum students.

The modest improvement in communication skills in the cohort who received professional development training is consistent with other findings. Utting and colleagues [20] found no improvement in observed communication skills following the introduction of more concentrated training, but suggested that students would need more time to implement and consolidate their new skills in order for improvements to be evident in video recorded consultations. Students in the present study were not regularly meeting patients on a one-to-one basis, and may have needed more opportunities to practise their skills for improvements to be established in their routine behaviour. This suggests that dedicated communication skills teaching may be required in addition to integrated teaching in order to promote observable improvements in clinical communication skills [7,8,29].

The findings showed that female students obtained higher scores for observed communication. This is in line with previous research indicating that female students tend to achieve higher grades in clinical communication tasks [30] than their male counterparts.

Students who were less nervous, and those who indicated that they were more confident about knowing how to listen to patients at the end of their second year, improved their communication skills more over time. This is in line with expectations. On the other hand, the absence of a relationship between patient-centred attitudes and observed behaviour warrants discussion. Previous research has shown that both a change in patient-centred attitudes and communication behaviour can be brought about [22]. Furthermore, previous research has found an increase in patient-centred attitudes in students receiving professional development training [24]. It is possible that, with little personal experience of working with patients, students find it difficult to integrate the abstract concept of 'patient-centredness' with their own developing skills.

The relationship between observed communication in the early years of the medical course and later OSCE scores was intriguing. It is important to acknowledge that the OSCE scores include marks for both practical clinical procedures and communication. This means that the skills assessed were broader than those taught within the 'professional development' component of the curriculum. Considering the impact of communication skills teaching on OSCE performance has the advantage of using a measure which is 'realistic', i.e. important to students and demonstrates the skills in context. However, the disadvantage is that students may not perform at their best when rushed, nervous, or keen to ensure that other practical skills are sufficiently highlighted. It is possible that the 'better communicators' of the traditional curriculum were hampered by these skills during the time-pressured OSCE assessments, or that the new curriculum teaching prepared students more effectively for OSCEs and allowed them to integrate their skills more appropriately. Alternatively, one could speculate that the students who joined the medical school when the new curriculum was introduced differed in some fundamental way from the previous cohorts. This difference may have affected the way they responded to the professional development teaching (i.e. which may explain the tendency for better scores in the videoed interviews in year 2) as well as their performance during the final clinical examination. Unfortunately, no definitive answer can be provided regarding these findings, and multi-method testing of communication skills may continue to be appropriate to assess how skills are demonstrated in isolation (in a simple simulated consultation) and when integrated (in a clinical examination)

The results of this study need to be viewed in the context of certain limitations, which primarily relate to the instruments employed. The Interview Rating Scale [17], which assesses basic communication skills without requiring medical knowledge, was deemed to be the best available scale at the inception of this research. However, the sensitivity and appropriateness of certain items appeared problematic, resulting in some items being excluded from analysis. Furthermore, there were issues concerning inter-rater reliability in using this measure [31]. This was dealt with as thoroughly as possible, by exploring inter-rater reliability using an appropriate method [28] and replacing the scores of one rater. Assessing the communication between students and simulated patients, although appropriate to the students' stage of training, may limit the generalisability of the findings to the clinical environment. In addition, each student only had the opportunity to conduct one simulated consultation at each time point in years 1 and 2, unlike the clinical examination, which assessed students' performance in several consultations. Finally, it should be recognised that this study was based on a small sample, from two cohorts at a single institution, which may limit the generalisability of the findings to other medical schools.

## Conclusions

The communication skills of medical students improved during the first two years of the course. The implementation of a curriculum including greater emphasis on professional skills and attitudes may have contributed towards an improvement in observed communication skills. While students' observed communication skills appeared independent of their patient-centred attitudes, students who were less nervous and reported a better understanding of how to listen to patients demonstrated better communication observable skills. Students may have benefited from more opportunities to practise basic communication skills on a one-to-one basis with patients to enable them to consolidate their professional learning and establish improvements in their routine behaviour.

## Competing interests

The authors declare that they have no competing interests.

## Authors' contributions

KJ performed the secondary video observations and the statistical analyses, and drafted the manuscript; LN participated in the conception, design and co-ordination of the study, data collection, statistical analyses, and drafting of the manuscript; AK contributed to the data collection and tabulation and performed secondary video observations; HP advised on statistical analysis and drafting of the manuscript; ML participated in the conception, design, coordination of the study and data collection. All authors read and approved the final manuscript.

## Pre-publication history

The pre-publication history for this paper can be accessed here:

http://www.biomedcentral.com/1472-6920/11/41/prepub
